# Enhanced emotional and motor responses to live versus videotaped dynamic facial expressions

**DOI:** 10.1038/s41598-020-73826-2

**Published:** 2020-10-08

**Authors:** Chun-Ting Hsu, Wataru Sato, Sakiko Yoshikawa

**Affiliations:** 1 Psychological Process Team, BZP, RIKEN, 2-2-2 Hikaridai, Seika-cho, Soraku-gun, Kyoto 619-0288 Japan; 2 Institute of Philosophy and Human Values, Kyoto University of the Arts, 2-116 Uryuyama Kitashirakawa, Sakyo, Kyoto, Kyoto 606-8271 Japan

**Keywords:** Human behaviour, Emotion, Social neuroscience

## Abstract

Facial expression is an integral aspect of non-verbal communication of affective information. Earlier psychological studies have reported that the presentation of prerecorded photographs or videos of emotional facial expressions automatically elicits divergent responses, such as emotions and facial mimicry. However, such highly controlled experimental procedures may lack the vividness of real-life social interactions. This study incorporated a live image relay system that delivered models’ real-time performance of positive (smiling) and negative (frowning) dynamic facial expressions or their prerecorded videos to participants. We measured subjective ratings of valence and arousal and facial electromyography (EMG) activity in the zygomaticus major and corrugator supercilii muscles. Subjective ratings showed that the live facial expressions were rated to elicit higher valence and more arousing than the corresponding videos for positive emotion conditions. Facial EMG data showed that compared with the video, live facial expressions more effectively elicited facial muscular activity congruent with the models’ positive facial expressions. The findings indicate that emotional facial expressions in live social interactions are more evocative of emotional reactions and facial mimicry than earlier experimental data have suggested.

## Introduction

Facial expressions of emotions are indispensable communicative signals that facilitate the establishment and maintenance of social relationships in real-life^1^. Studies have shown that human beings prioritize attention to faces over other forms of visual input^2^. Moreover, emotional facial expressions, when compared with neutral expressions, accelerate attentional engagement and delay attentional disengagement^3^. Attention to emotional facial expressions supports the understanding of others’ emotional expressions and intentions in social interaction.

During social interaction, the observation of facial expressions automatically elicits emotional arousal and spontaneous facial mimicry^4–6^, as reflected by increased zygomaticus major (ZM) activity when viewing happy facial expressions and increased corrugator supercilii (CS) activity when viewing angry facial expressions. Specifically, the observation of dynamic emotional facial expressions elicits stronger subjective arousal experiences and more evident facial mimicry, compared with observation of static facial expressions^7,8^. Automatic mimicry may facilitate emotion perception or recognition via an embodied mechanism^9,10^. It also enhances interpersonal synchronization^11^, which further facilitates emotion contagion^12–14^, empathy^15,16^, and social interaction^17,18^.

Most psychological studies regarding social interaction have used highly controlled laboratory settings with prerecorded static photographs or video clips that differed from naturalistic social behaviors in aspects such as visual fidelity, social context, and the potential for social interaction with the stimulus material. Experts have called for a reconsideration of the ecological validity of these stimuli and of the design of psychological studies of non-verbal and verbal interpersonal communication and interactions, which may improve the interpretability and generalizability of such findings^19,20^. To the best of our knowledge, only one study has compared the effects of live interaction with those of prerecorded stimuli in terms of emotional and motor responses to facial expressions. In that study, the researchers designed a liquid crystal (LC) shutter system that switched between transparent and opaque to control the timing of participants’ exposure to models who were making eye contact or averting their gaze in real time^21^. Their findings indicated that differences in ZM activity while viewing a static smiling versus a neutral facial expression were observed only when the participants believed that they were being watched by the model^22^. However, the implications of their EMG results were ambiguous because the ZM responses to smiles did not differ between conditions with and without beliefs about being watched. Instead, the attenuation of ZM responses to neutral expressions caused the differences. Therefore, increased facial mimicry when participants believed that they were being watched could not be concluded. Furthermore, the study used static (rather than dynamic) facial expressions as stimuli, despite previous studies’^7,8^ suggestions that dynamic facial expressions would be more effective for investigating the effects of live interactions on subjective emotion perception and facial mimicry.

**Figure 1:**
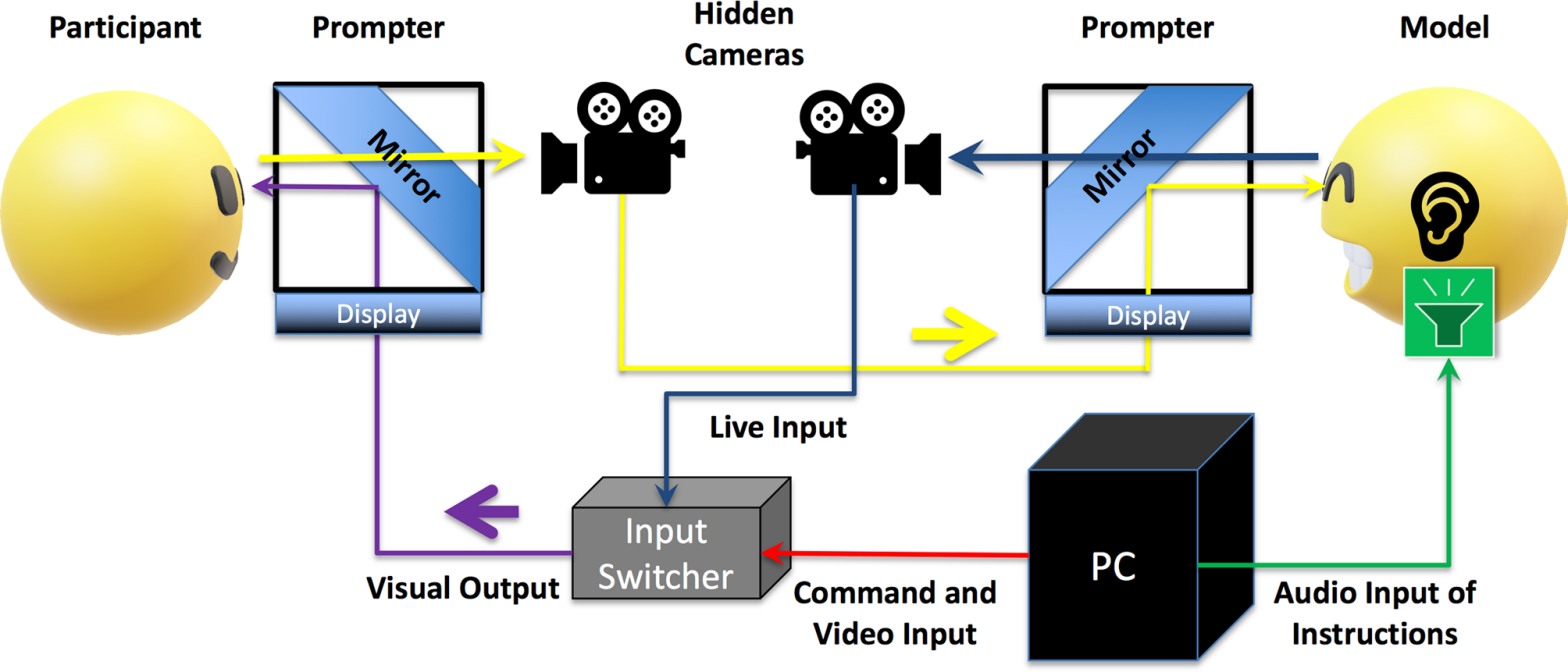
The experimental facilities. The participant and model each faced a prompter. The prompter consisted of a horizontally positioned display and an obliquely positioned transparent mirror. The participant could view the visual information on the display via the reflection in the mirror, while the participant’s image was video-recorded and live-relayed by a camera hidden behind the mirror. The participant’s image was then sent to the to the prompter of the model (the yellow route). The image of the model captured by the hidden camera was sent to the input switcher (the blue route) as one of the visual output options. The PC running the paradigm sent commands (the red route) through a serial port to the input switcher to determine the visual output for the participant to view (the purple route). During video trials, the PC sent the instructions and prerecorded video stimulus (red) to the input switcher, and the switcher sent only the input from the PC to the participant’s prompter (purple). During live trials, the switcher first relayed the instructions from the PC to the participant (red), and the PC then sent the audio signal via Bluetooth earphones to the model to instruct the model to produce dynamic facial expressions (the green route) while sending commands (red) to the input switcher to switch to the model’s live image input (blue) and to switch back after the duration of the stimulus (3 s).

**Figure 2:**
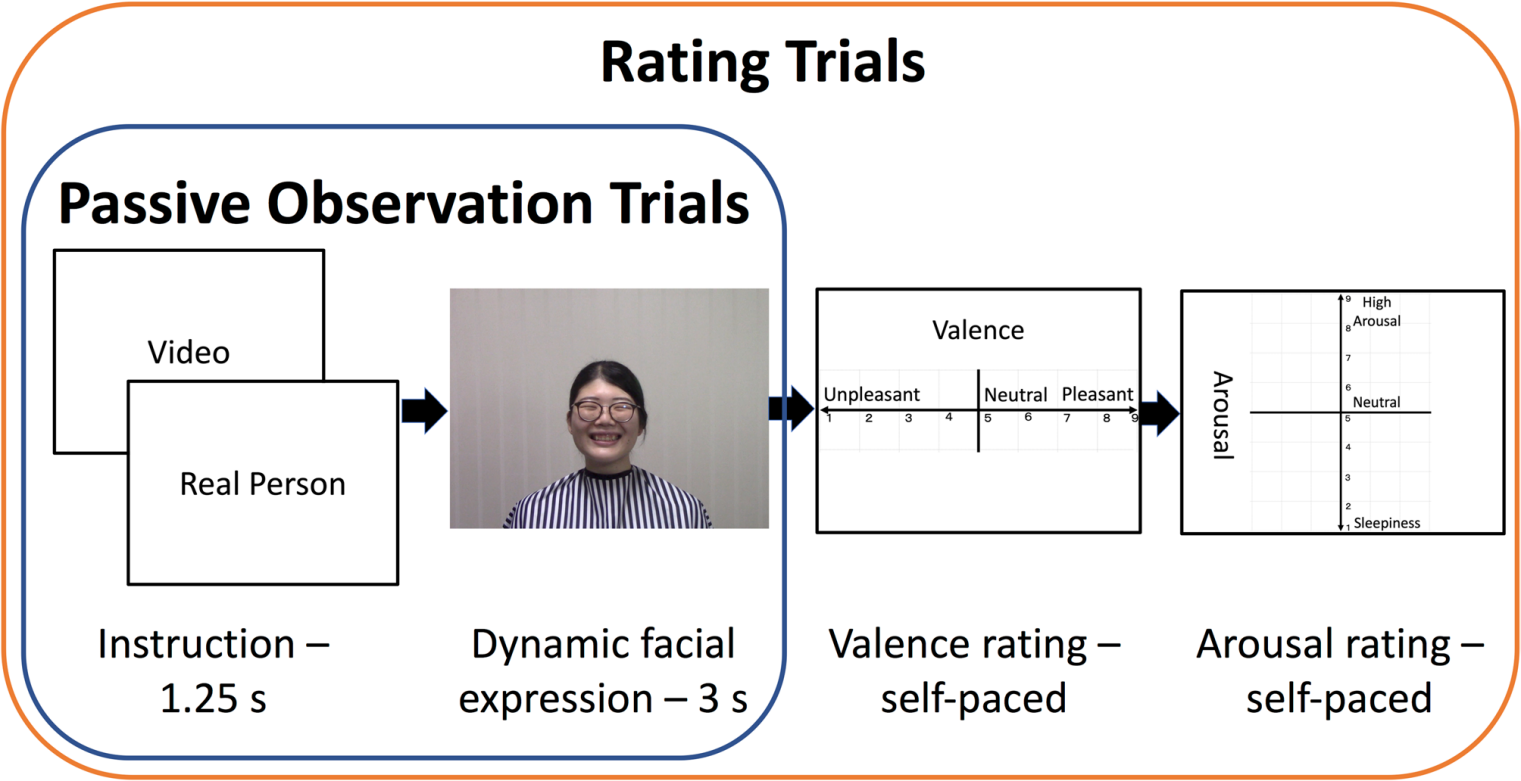
The procedure of each trial. During the passive observation trials, the participant first saw the instruction for either “Video” or “Real Person,” followed by 3 s of prerecorded or live dynamic facial expression, with the fixation cross lasting for the duration of the jittered inter-stimulus interval. Later in the rating trials, the participants saw the instruction followed by the stimulus as in the passive observation trials, and were asked to rate the valence and arousal using the keyboard in front of them.

In the present study, we tested the effect of live interaction using dynamic facial expressions on subjective emotion and facial mimicry. We designed a video camera relay system to allow participants to view either models’ live or prerecorded dynamic facial expressions (Fig. 1). Variable mimicry responses and emotion contagion across genders have been widely reported^23–25^. Thus, to avoid confounding factors, we recruited only female participants and models. Following a 3-min conversation with the model using the video relay system, each participant passively viewed 60 prerecorded or live videos of positive (smiling) or negative (frowning) dynamic facial expressions, while ZM and CS EMG activity was recorded to estimate the magnitude of spontaneous facial mimicry. The participants then completed 16 rating trials that assessed dimensions of valence and arousal^26^ (Fig. 2). Considering that arousal, social attention, and pro-social behavior are enhanced in live social interaction^27,28^, we expected to observe an interaction between the presentation condition (i.e., live vs. video) and emotion (i.e., positive vs. negative), such that subjective emotion perceptions and spontaneous facial mimicry would be more prominent under the live conditions.

## Results

### Subjective ratings

**Figure 3:**
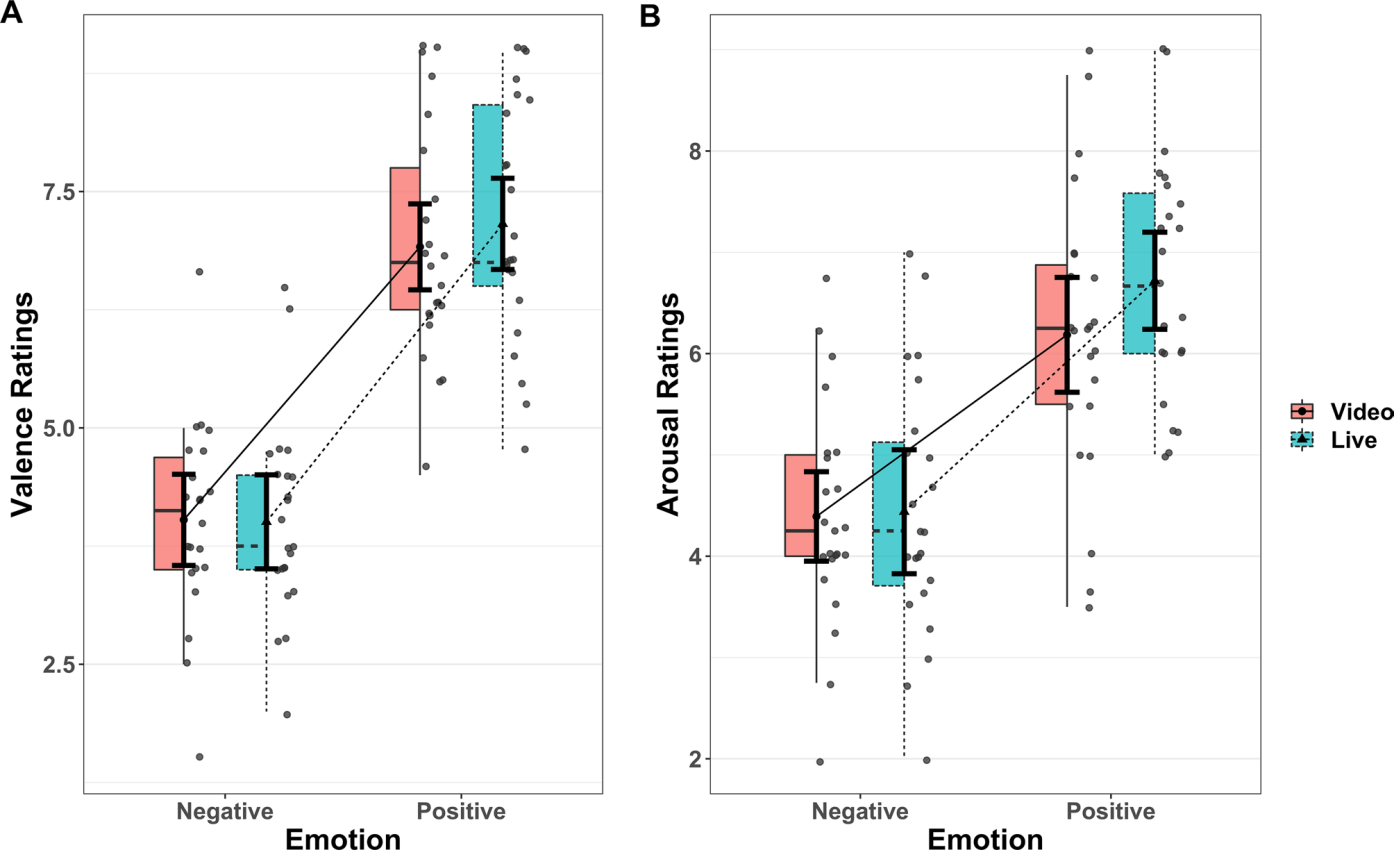
Subjective ratings. For each condition, the right half shows the scattered dots of participant-wise mean values. The group mean value is shown with a filled dot (video condition) or triangle (live condition) in the middle, accompanied by error bars indicating the within-subject standard error. The box on the left half defines the median, along with the first and third quartiles of the distribution; the upper or lower whiskers extend from the hinge to the most extreme value no further than 1.5 * IQR from the hinge. Panel A: valence. Panel B: arousal. A significant interaction was observed between emotion (positive, negative) and presentation condition (video, live). Follow-up simple effect analysis revealed higher valence and arousal ratings under the positive-live condition than under the positive-video condition, but no differences were observed between the negative conditions.

A linear mixed effects (LME) model was used to analyze subjective valence and arousal ratings (Fig. 3). Fixed effects included emotion (positive and negative) and presentation condition (live and video) and the interaction term. Random effects included by-subject random intercepts and random slopes for the effect of emotion and presentation condition to account for the within-subjects design, which was determined by means of model comparisons.

Valence ratings included a significant main effect of emotion in which positive was rated higher than negative conditions and the interaction between emotion and presentation condition (Table 1). Planned simple effect analysis showed higher valence ratings for the positive-live than for the positive-video condition (estimate = 0.243, SE = 0.119, df = 46, t = 2.052, two-tailed *p* = 0.0459), but no difference between the negative-live and negative-video conditions (estimate = −0.021, SE = 0.0121, df = 47.2, t = 0.174).

**Table 1:** Statistical summary of valence ratings.

Effect	Beta	95% CI	SE	df	t-value	Pr( >|t|)	Rsq	
**Fixed effects**								
Intercept	5.527	(5.256, 5.798)	0.132	22.821	41.784	< 2e−16		
Emotion	2.135	(1.607, 2.664)	0.259	23.066	8.257	2.43e−08	0.748	
Presentation	0.0786	(− 0.0641, 0.221)	0.0698	23.795	1.126	0.2714	0.052	
Interaction	0.132	(0.00669, 0.257)	0.0637	285.27	2.076	0.0387	0.015	

Group	Effect	Variance	SD	SD 95% CI	Corr. I	Corr. 95% CI	Corr. E	Corr. 95% CI
**Random effects**								
Subject	Intercept	0.379	0.616	(0.462, 0.861)				
	Emotion	1.491	1.221	(0.928, 1.693)	0.24	(− 0.189, 0.589)		
	Presentation	0.0655	0.256	(0.139, 0.405)	− 0.04	(− 0.559, 0.466)	0.04	(− 0.463, 0.527)
Residual		0.339	0.582	(0.537, 0.634)				

CI: confidence interval; Corr. I.: correlation with the random effect of intercept; Corr. E.: correlation with the random effect of emotion; df: Satterthwaite approximations to degrees of freedom; Rsq: effect size of semi-partial R-squared; SD: standard deviation; SE: standard error.

Arousal ratings also included a significant main effect of emotion, with positive conditions rated as more arousing than negative ones; a trend for the main effect of presentation condition, with live rated as more arousing than video conditions; and the significant interaction between emotion and presentation condition (Table 2). Simple effect analysis showed higher arousal ratings for the positive-live than for the positive-video condition (estimate = 0.534, SE = 0.0167, df = 33.5, t = 3.203, two-tailed *p* = 0.003), but no difference between negative-live and negative-video conditions (estimate = 0.046, SE = 0.0167, df = 33.8, t = 0.277).

**Table 2:** Statistical summary of arousal ratings.

Effect	Beta	95% CI	SE	df	t-value	Pr( >|t|)	Rsq	
**Fixed effects**								
Intercept	5.434	(5.160, 5.707)	0.134	23.1	40.617	< 2e−16		
Emotion	1.44	(0.863, 2.017)	0.282	23.094	5.105	3.55e−05	0.531	
Presentation	0.205	(− 0.012, 0.423)	0.106	23.106	1.93	0.066	0.139	
Interaction	0.244	(0.117, 0.371)	0.065	266.24	3.770	0.000201	0.051	

Group	Effect	Variance	SD	SD 95% CI	Corr. I	Corr. 95% CI	Corr. E	Corr. 95% CI
**Random effects**								
Subject	Intercept	0.388	0.623	(0.468, 0.869)				
	Emotion	1.782	1.335	(1.017, 1.849)	0.14	(− 0.281, 0.518)		
	Presentation	0.212	0.46	(0.324, 0.666)	− 0.33	(− 0.678, 0.128)	− 0.41	(− 0.724, 0.026)
Residual		0.341	0.584	(0.538, 0.638)				

CI: confidence interval; Corr. I.: correlation with the random effect of intercept; Corr. E.: correlation with the random effect of emotion; df: Satterthwaite approximations to degrees of freedom; Rsq: effect size of semi-partial R-squared; SD: standard deviation; SE: standard error.

### Facial EMG

**Figure 4:**
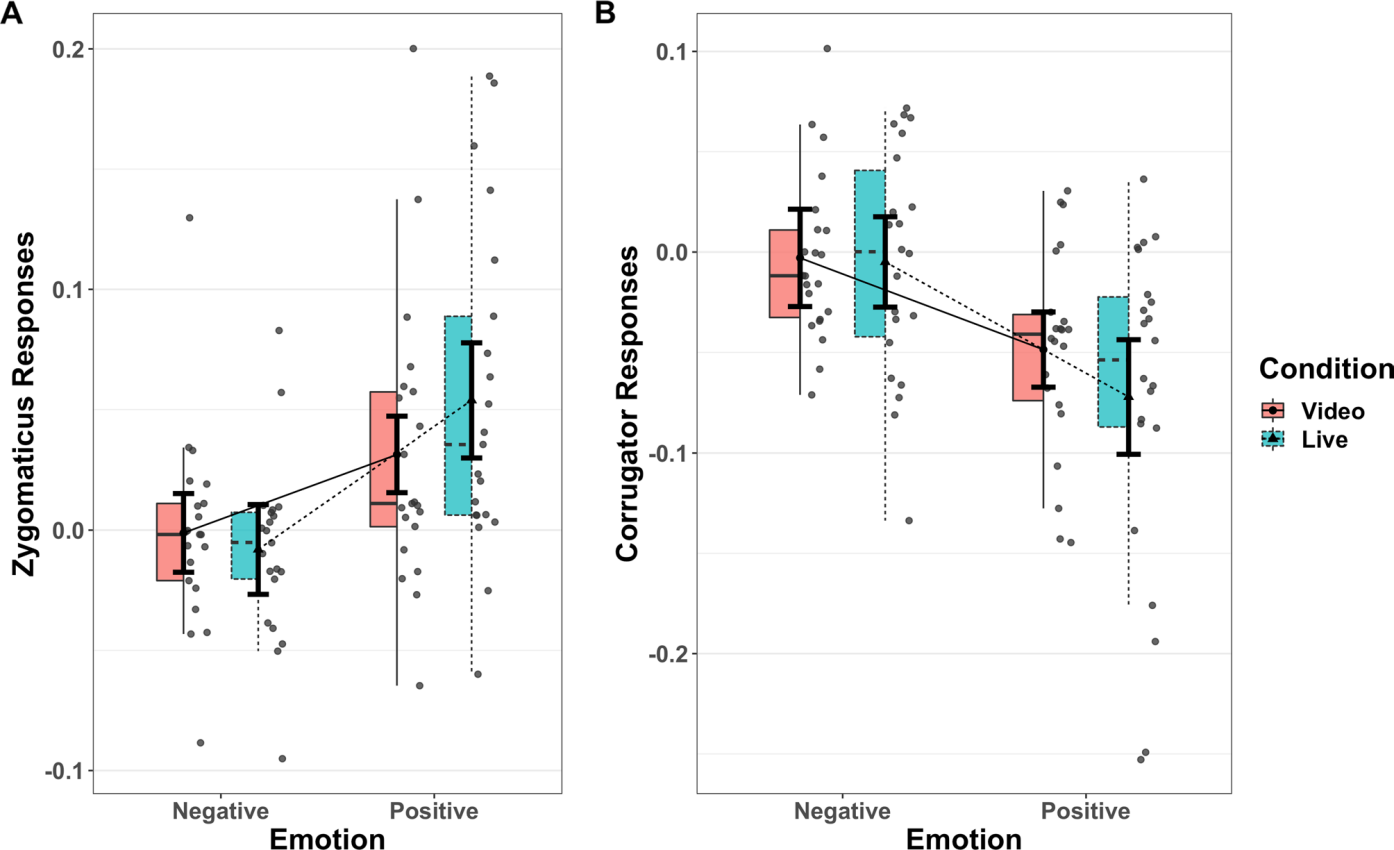
Facial electromyography (EMG) results. Please see the legend of Fig. 3 for component descriptions of the boxjitter plots. Panel A: zygomaticus major (ZM). Panel B: corrugator supercilii (CS). The EMG results showed a significant interaction between emotion (positive, negative) and presentation condition (video, live), which consisted of significant differences between the positive-live and positive-video conditions, but not between the negative conditions.

The log-transformed absolute values of the ZM and CS activity were used to calculate differences between effects of the neutral phase (0–1 s after stimulus onset) and the maximal phase (2.5–3.5 s after stimulus onset) of the dynamic facial expression, which were used as the dependent variable (Fig. 4). The same LME model as that used for the ratings was applied for both ZM and CS.

The ZM also showed a significant main effect of emotion, with greater muscle activation under the positive conditions than under the negative conditions as well as an interaction between emotion and presentation condition (Table 3). Simple effect analysis showed greater muscle contraction in positive-live than in positive-video condition (estimate = 0.0225, SE = 0.00074, df = 67.7, t = 3.024, two-tailed *p* = 0.0035), but no difference between the negative-live and negative-video conditions (estimate = −0.0069, SE = 0.0074, df = 68.2, t = −0.929).

**Table 3:** Statistical summary of zygomaticus major reactions.

Effect	Beta	95% CI	SE	df	t-value	Pr( >|t|)	Rsq	
**Fixed effects**								
Intercept	0.019	(0.002, 0.036)	8.201e−03	20.71	2.319	0.031		
Emotion	0.0334	(0.014, 0.053)	9.490e−03	21.01	3.52	0.00203	0.371	
Presentation	0.0055	(− 0.003, 0.014)	3.874e−03	19.45	1.421	0.17121	0.088	
Interaction	0.0147	(0.005, 0.024)	4.824e−03	1053	3.041	0.00242	0.009	

Group	Effect	Variance	SD	SD 95% CI	Corr. I	Corr. 95% CI	Corr. E	Corr. 95% CI
**Random effects**								
Subject	Intercept	0.00129	0.036	(0.028, 0.045)				
	Emotion	0.00164	0.041	(0.029, 0.046)	0.54	(0.089, 0.539)		
	Presentation	7.06e−05	0.008	(0.006, 0.020)	− 0.28	(− 0.282, − 0.281)	0.55	(− 0.738, 0.549)
Residual		0.00643	0.08	(0.079, 0.081)				

CI: confidence interval; Corr. I.: correlation with the random effect of intercept; Corr. E.: correlation with the random effect of emotion; df: Satterthwaite approximations to degrees of freedom; Rsq: effect size of semi-partial R-squared; SD: standard deviation; SE: standard error.

The CS showed a main effect of emotion, with the CS less active under the positive conditions than under the negative conditions, as well as an interaction (Table 4). Simple effect analysis showed less activity under the positive-live condition than under the positive-video condition (estimate = −0.024, SE = 0.0096, df = 51.3, t = −2.467, two-tailed *p* = 0.017), but no difference between the negative-live and negative-video conditions (estimate = −0.002, SE = 0.0096, df = 50.6, t = −0.212). In addition, the by-subject random slope of the presentation condition exhibited a clear positive correlation with the random slope of emotion (Table 4, 95% CI = 0.455, 0.474).

**Table 4:** Statistical summary of corrugator supercilii reactions.

Effect	Beta	95% CI	SE	df	t-value	Pr( >|t|)	Rsq	
**Fixed effects**								
Intercept	− 0.0322	(− 0.048, − 0.017)	7.641e−03	21.06	− 4.208	0.000393		
Emotion	− 0.0399	(− 0.065, − 0.015)	1.220e−02	22.03	− 3.273	0.00347	0.328	
Presentation	− 0.0090	(− 0.021, 0.002)	5.572e−03	24.06	− 1.623	0.117718	0.109	
Interaction	− 0.0108	(− 0.021, − 0.00024)	5.367e−03	1137	− 2.006	0.045077	0.004	

Group	Effect	Variance	SD	SD 95% CI	Corr. I	Corr. 95% CI	Corr. E	Corr. 95% CI
**Random effects**								
Subject	Intercept	0.00112	0.0335	(0.0335, 0.0336)				
	Emotion	0.00295	0.054	(0.052, 0.056)	0.377	(0.3769, 0.3771)		
	Presentation	0.000367	0.019	(0.011, 0.030)	0.99	(0.707, 1.001)	0.47	(0.455, 0.474)
Residual		0.008469	0.092	(0.092, 0.093)				

CI: confidence interval; Corr. I.: correlation with the random effect of intercept; Corr. E.: correlation with the random effect of emotion; df: Satterthwaite approximations to degrees of freedom; Rsq: effect size of semi-partial R-squared; SD: standard deviation; SE: standard error.

### Live and prerecorded stimuli validation

We asked a separate group of naïve participants to rate the valence and arousal of a subset of prerecorded and live video clips, without knowing whether the clip showed a live performance. The LME analysis and model comparison showed significant emotion effect in valence and arousal ratings, and significant presentation condition effect in valence ratings (live performances were rated as more negative), but the fixed effect of interaction was no longer statistically significant (Supplementary Table S1, S2, Fig. S1); model comparison favored the model without the fixed effect of interaction (valence: *Χ*^2^ = 0.0426, two-tailed *p* = 0.836; arousal: *Χ*^2^ = 0.8648, two-tailed *p* = 0.352). A Bayesian paired t-test also showed moderate supporting evidence for the null hypothesis, i.e., no difference between the subject-wise (positive-live minus negative-live) and (positive-prerecorded minus negative-prerecorded) (for valence and arousal ratings: BF_10_ = 0.211, BF_01_ = 4.73, see Supplementary Table S3 for statistical details and frequentist statistics). When evaluating/guessing whether a clip is prerecorded or a live performance, there was moderate supporting evidence for the null hypothesis that participants’ accuracy rates did not differ from the level expected by chance (BF_10_ = 0.214, BF_01_ = 4.679, see Supplementary Table S4 for statistical details and frequentist statistics).

## Discussion

In both the rating and EMG data, we observed a significant main effect of emotion in a pattern that was consistent with those of earlier studies^7,8^. In the rating data, we observed higher valence and arousal ratings for the positive conditions. In the EMG data, we observed enhanced ZM and reduced CS activity under the positive smiling condition. The consistent pattern confirmed the validity of our stimuli showing dynamic emotional facial expressions.

More importantly, we observed significant interactions between live conditions and emotion in both the rating and EMG data. The interactions showed a consistent pattern, in which positive smiling expressions were rated as more arousing and more positive under live conditions; the ZM contracted more and the CS was more relaxed under the positive-live conditions than under the positive-video conditions. The effect of dynamic facial expressions on subjective ratings and spontaneous facial mimicry was more pronounced when the stimuli were live than when they were prerecorded. The validation rating study of randomly selected prerecorded and live performance video clips showed that such an effect is unlikely due to the model performing more enthusiastically during live performance trials.

Our findings corroborate those of Hietanen et al.^22^, who also reported greater ZM activation when participants viewed smiling expressions, compared to neutral facial expressions, if they believed that they were being watched. However, that study presented only static and not dynamic facial expressions; only smiling faces were included in the stimuli. Furthermore, their EMG results were not conclusive because the differences mainly consisted of attenuated ZM activity when participants viewed neutral facial expressions while believing that they were being watched by the model; ZM responses to smiles did not vary respect to participants’ beliefs about being watched. Therefore, to the best of our knowledge, the present study is the first to offer evidence that live social interaction causes greater enhancement of facial mimicry of dynamic facial expressions, compared to prerecorded video presentations.

While our results clearly demonstrate the facilitative effect of live interaction on emotional and motor responses to dynamic facial expressions, the underlying factors remain unclear. Below, we discuss possible underlying factors from the metacognitive (e.g., belief/knowledge-induced) and stimulus-driven (e.g., detection of microexpressions) perspectives. We first consider the socially facilitative properties of live interaction. Social psychological theories have long been proposed to explore the possible mechanisms by which the presence of others changes behavior; for example, the “Audience Effect” has been thoroughly reviewed by Hamilton and Lind^29^. In the context of social facilitation, Zajonc used drive theory to explain how the presence of conspecifics increased individuals’ arousal and influenced their performance of tasks depending on the nature of the task^30,31^. Bond attributed social facilitation to the performer’s active regulation of self-representation (i.e., public image) and suggested that the loss of public esteem from poor public performance would cause embarrassment and exacerbate social impairment^32^. Tennie et al. further elaborated on the motivation for reputation self-management. To function effectively, social interaction depends largely on mutual trust; a good reputation makes an individual more trustworthy, gives them an advantage in partner choices, and the possibility of engaging with an individual of good standing provides an incentive for others to cooperate^33^.

In the social top-down response modulation (STORM) theory, Wang and Hamilton specifically proposed that mimicry is a strategic intervention that aims to change the social world for self-advancement. Earlier studies have indeed demonstrated that mimicry has positive consequences in terms of social interaction. Mimicry leads to increased liking and perceived closeness toward the mimicker, facilitating pro-social behavior^34–36^; it functions as a “social glue” that helps to establish social rapport^37,38^. Through associative learning or evolutionary hardwiring^39^, human beings display more pro-social behavior, including facial mimicry, when they know that they are being watched. The role of associative learning has been emphasized by appeals to the fact that facial mimicry considers learned social context and knowledge^40,41^. For example, a low-power individual is likely to respond by smiling to any expression from a higher-power person^42^. Frith further highlighted the role of implicit metacognition in social interaction^43^. It enables us to adopt a “we-mode” through which we mentalize and automatically consider the knowledge and intentions of others. In the present study, we invited only models and participants of the same gender and age and allowed them to socialize harmoniously before the experiment to control for the effects of social context. It is important to further investigate the effects of social context in future studies using the present live interaction paradigm.

Although some theories of social facilitation have emphasized the importance of metacognitive or top-down modulation, evidence that spontaneous facial mimicry is a result of non-conscious perception of facial expressions has also been presented^5,44^. In real-life social interaction, circumstances often arise in which an individual strives to suppress or conceal their genuine emotion, resulting in microexpressions that last 1/25–1/5 seconds^45–47^. Research regarding microexpressions has focused on their applications in clinical diagnosis, forensic investigation, and security systems^48^; it is important to investigate whether, in live interaction, the detection of microexpressions could also elicit facial mimicry and emotion contagion^49,50^. Such evidence could support the alternative bottom-up component of the mechanisms of emotion contagion in social interactions. In the present study, we could not clearly differentiate between metacognitive (e.g., belief/knowledge-induced) and stimulus-driven (e.g., detection of microexpressions) components of facial mimicry and emotion contagion. However, our validation study showed that without the prior knowledge of whether the video clip is a live performance, the interaction effect no longer exists. This provided additional information that effects observed in the current study were more likely to be affected by metacognitive components of emotion contagion. Future studies with more realistic designs may yield further insights regarding this aspect.

We found only increased ZM activity in the positive-live condition compared with the positive-video condition; we found no difference in CS activity between negative-live and negative-video conditions. Several factors may explain this difference. First, it is compatible with earlier findings regarding social interaction, in which frowning is not mimicked in the context of anger because anger is not an emotion that facilitates affiliation between people^17,40^, which further supports the interpretation of mimicry and social facilitation. Second, at the beginning of the experiment, each participant had 3 min of friendly interaction with the model through the video relay system. This affiliation-building atmosphere may have reduced the likelihood that participants would mimic negative facial expressions during the experiment. Third, the negative expression (frowning) performed by the models in both the video and live situations was not particularly intense. This situation was reflected in the arousal ratings, which were significantly lower for the negative conditions than for the positive conditions. The lack of intensity in the models’ performances may also have weakened negative emotion contagion.

Finally, while our sample size was insufficient to investigate the effect of individual differences, CS responses showed a positive correlation between the by-subject random slope of presentation conditions and the by-subject random slope of emotion. This indicated that participants who responded more strongly to the emotion conditions, also responded more to the manipulation of presentation conditions. In future studies, it will be important to investigate whether individual differences in personality traits would modulate the effect of live interaction in emotion contagion.

In conclusion, by using a live-relay prompter system to present prerecorded and live images, we showed that subjects’ perception and spontaneous facial mimicry of dynamic facial expressions were stronger under the live conditions than under videotaped conditions. Our paradigm was shown to be valid with respect to the balance between real-life social experiences and well-controlled laboratory settings. Variations based on this design could be used to further investigate the effects of individual differences, as well as social cognitive variables such as gender, power imbalances, and social contexts and to differentiate between metacognitive versus stimulus-driven components of emotion contagion.

## Methods

### Participants

Twenty-three female adults were recruited (mean age ± SD = 22.48 ± 2.27 years, range 18–27 years) in the city of Kyoto. The required sample size was determined by a priori power analysis using G*Power software ver. 3.1.9.2^51^, based on a previous study that had tested the effects of dynamic versus static facial expressions on subjective emotional ratings. As an approximation of the present analyses, we assumed a two-step procedure that included estimating the individual average values and conducting within-subjects analysis of variance. An F-value of 0.25 (medium-sized effect), an α level of 0.05, and power (1 − β) of 0.80 were assumed. The results of the power analysis showed that more than 21 participants were needed. The study was approved by the Ethics Committee of the Unit for Advanced Studies of the Human Mind, Kyoto University, and was performed in accordance with the ethical standards of the committee. Written informed consent was obtained from all participants prior to their participation in the study. All participants consented to publication, because video recording was carried out in this study. All participants were rewarded monetarily.

### Material

Two female models, both 20 years of age, were employed. Each model recorded at least 20 video clips of positive and negative dynamic facial expression. Both models provided written informed consent for their identity-revealing images, which appeared in Fig. 2, to be used for scientific publications. The positive facial expression consisted of smiling, and the negative expression consisted of frowning. The models wore an apron covering their clothes when recording the video clips and during the experiments. They were asked to use hair pins to maintain their hairstyles consistent between the recorded video clips and the live stimuli. The models faced the camera frontally. The clips lasted three seconds, comprising one second of neutral expression, one second of gradual dynamic change, and one second of sustained maximal emotional facial expression. Fifteen positive and fifteen negative clips were used in the passive viewing part of the experiment, two of each condition were used for the rating practice, and four of each condition were used for the actual ratings. No clips were repeated within a part.

### Facilities

The model and the participant each faced one prompter, which consisted of a mirror reflecting a TV screen and a Canon VIXIA HF R800 camera concealed behind the mirror (Fig. 1). The model was able to view the live-relay image of the participant at all times. The input from the participant’s prompter was controlled by an Imagenics SL-41C Switcher, which received serial port signals from the Precision T3500 with Windows 7 Professional and Presentation (Neurobehavioral Systems) software running the experimental paradigm and switched between the visual input from the PC and the model’s camera. The models wore the same apron in the prerecorded clips and wore a Pasonomi TWS-X9 Bluetooth earphone that received sound signals from the Presentation PC that instructed them to produce dynamic facial expressions. The original resolution of the video clips and live-relay images from the Canon VIXIA HF R800 camera was 1920 × 1080. The Presentation software outputs the video clips at a resolution of 1280 × 960, cropping the images’ flanks; the screens that the participant faced were also set to 1280 × 960 resolution to ensure consistency across the prerecorded video and live-relay images. The participants were fitted with electrodes to record EMG data from the ZM and CS, which were transmitted to the BrainAmp ExG MR amplifier and saved by the BrainVision Recorder software.

### Paradigm and procedure

The study design involved two crossed factors: presentation condition (video vs. live) and emotion (positive vs. negative), resulting in four conditions. Each participant only interacted with one of the models. Upon arrival, the participant was debriefed with respect to informed consent and signed the consent form. Then, the model attached the EMG electrodes to the participant and sat down in front of her prompter. To demonstrate to the participant that the prompter could deliver live relay of the model’s image and to allow the participant to become familiar with the model, the participant and the model engaged in 3 min of conversation about school, work, food, and the weather through the prompter system. The first part of the experiment consisted of passive viewing. The participant fixated on the cross in the center of the screen (jittered between 2000 and 3750 ms, mean inter-trial interval was 2604 ms) until the instruction screen showed either “Video” or “Real Person” for one second. During the video trials, one of the prerecorded video clips followed immediately after the instruction. During the live trials, the models heard the signal through their earphones as the instruction screen was shown, and a change in pitch signaled the model to perform the correct dynamic facial expression. The screen showed the fixation cross again after the facial expressions had been shown. The participant first performed eight practice trials, two trials under each condition. Afterwards, participants performed 15 passive viewing trials under each condition, for a total of 60 trials, with a break after 32 trials. For each participant, the sequence of conditions at the trial level was pseudo-randomized to ensure that there were no more than three sequential trials of the same emotion (positive or negative) or presentation condition (prerecorded or live). For the prerecorded positive and negative conditions, the presentation sequence of the 16 videos of the same condition was randomized. EMG data were collected during the passive viewing trials.

The second part involved affective grid ratings^26^. The following instruction was provided to the participants: “When you see the images, please rate the emotion that you feel.” The participants were then instructed to rate the valence (pleasant vs. unpleasant) and arousal (low vs. high arousal) according to their feeling on Likert scales ranging from 1 to 9 using the keyboard in front of the prompter. Each trial began in a manner similar to the passive viewing component, and the dynamic facial expressions were followed by valence and then arousal ratings (Fig. 2). Participants first performed eight practice rating trials (two trials per condition) and then 16 test rating trials. Finally, the electrodes were removed, and the participant completed the autistic spectrum quotient and interpersonal reactivity index questionnaires, which were not analyzed in the current study. The participant was then monetarily rewarded and dismissed.

### Live stimuli validation

Both models’ live performances in the passive viewing trials were video-recorded and visually inspected to ensure that dynamic facial expressions were performed correctly. One positive trial for two participants and one negative trial for two other participants were excluded because the dynamic facial expressions were not performed correctly or were performed with obvious delay, leaving 1376 trials of EMG data for analysis.

To ensure that the observed interaction between emotion and presentation condition was not due to enhanced emotional expression during live performances, an online rating study was designed. Thirty-two prerecorded video clips (8 per emotion condition—positive or negative, per model) and 32 randomly selected clips of live performances were presented to naïve participants. The following hypothetical context was provided: “Ms. A and B attended an audition for a play. The director wanted to see them perform smiling and frowning expressions. They were recorded making the expressions when the director was not in the room, and also when they performed live in front of the director.” For each stimulus, participants were asked to rate the valence and arousal using the same instruction as in the live-interaction experiment. They were then asked to judge whether they were watching a prerecorded video or a live performance. Data were collected from 25 participants (8 females, mean age ± SD = 24.68 ± 6.57, range 18–51 years).

### EMG data preprocessing

Raw EMG data were preprocessed using the Toolbox EEGLAB v.2019.0 on MATLAB. The data were screened for movement artifacts, then a notch filter was applied around 60 Hz and at multiples of 60 Hz, a high-pass filter was applied at 20 Hz, and a low-pass filter was applied at 500 Hz. For each trial, the signal was detrended and the baseline was removed. Baseline was defined as the mean value of 3 s prior to stimuli onset until 1 s after the stimuli onset, which was the end of the model’s neutral expression, immediately prior to the dynamic facial expression change. All data points in the trial, 5000 Hz for 8 s, from 3 s prior to the stimulus onset to 5 s after the stimulus onset, were subtracted from the baseline value. For each signal, the absolute values plus one was natural log-transformed to correct for the right-skewness of the raw data distribution. This transformation is commonly applied to EMG data to reduce the impact of extreme values^13,52–55^. The log-transformed data were used in the main text to show results in a manner comparable to the approach used in previous studies. However, the statistical analyses of non-transformed data are shown in the supplementary information (Supplementary Text, Table S5 and S6 and Fig. S2). They show similar patterns, while the assumption of residual normality in the LME models was not as strongly satisfied.

### Statistical analyses of live interaction data

LME models, model comparisons and influence diagnostics were performed in R (v4.0.2) using the packages lmer4 1.1-23, lmerTest 3.1-2, HLMdiag 0.3.1, and emmeans 1.4.6, and optimizer BOBYQA. Dependent variables included valence ratings, arousal ratings, EMG difference (ZM and CS) between the neutral (0–1 s after stimulus onset) and maximal phases (2.5–3.5 s after stimulus onset) of the dynamic facial expression. For each LME model, emotion and presentation condition was considered as fixed effects, while the subject was included as a random factor. Model complexity in terms of fixed and random effects was increased in a stepwise fashion; each step was compared with the less complicated model using F-tests in lmerTest::anova() until the more complex model was no longer significantly preferable to the simpler model. The optimal models were described by the formula Y ~ 1 + emotion * presentation_condition + (1 + emotion + presentation_condition | subject). As fixed effects, emotion and presentation condition were entered with the interaction term into the model. As random effects, random intercepts for subjects were included, as were by-subject random slopes for the effect of emotion and presentation condition.

For model criticism^56^, upward residual and influence analysis was performed using the HLMdiag package^57^. At the trial level, trials were excluded with an absolute value of the standardized residual larger than three, or with a Cook’s distance larger than 1.5 × the interquartile range above the third quartile (*Q*_*3*_ + 1.5 × *IQR*). After the reduced data had been re-fitted, the Cook’s distance was checked to exclude highly influential participants. In total, the following trials were excluded: 22 (5.98%) of valence rating, 35 (9.51%) of arousal rating, 195 (14%) of CS data (including all data from participant 14), and 267 (19%) of ZM data (including all data from participants 8 and 14). Participant 14 had very high CS activity in negative conditions; both participants 8 and 14 had very high ZM activity in positive conditions.

Satterthwaite’s estimation of degrees of freedom is reported. Significant interactions were followed by a planned simple effect analysis of calculating the difference between the live and video conditions in each emotion category. Effect size estimation of the semi-partial R-squared (variance explained) of fixed effects was carried out using r2glmm 0.1.2 with the Kenward–Roger approach^58–60^. Boxjitter plots were created with packages afex 0.27–2, ggplot2 3.3.0, ggpol 0.0.6 and cowplot 1.0.0. Two-tailed statistical *p *values are reported throughout the text.

### Statistical analyses of validation rating data

To determine whether the fixed effect of interaction arose in the validation data, for valence and arousal ratings, LME and model diagnostics were performed using the same formula: Y ~ 1 + emotion * presentation_condition + (1 + emotion + presentation_condition | subject), as described in the section “Statistical analyses of live interaction data.” Model comparisons were also carried out with the LME models without the fixed effect of interaction between emotion and presentation condition, using F-tests in lmerTest::anova(). A Bayesian paired sample t-test was also performed for subject-wise values of (positive-live minus negative-live) versus (positive-prerecorded minus negative-prerecorded) using JASP 0.13.1. For the evaluation of prerecorded versus live performances, the subject-wise accuracy rate was tested against the 0.5 chance level using a Bayesian one-sample t-test. The default Cauchy prior width of 0.707 was used.

## Supplementary information

Supplementary Information.
