# Supplementary material for: Enhanced emotional and motor responses to live versus videotaped dynamic facial expressions
Source: Sci Rep. 2020 Oct 8;10:16825. doi: 10.1038/s41598-020-73826-2 (PMC7544832; doi:10.1038/s41598-020-73826-2)
Supplement: Supplementary file 1 — Supplementary Information. [file 41598_2020_73826_MOESM1_ESM.docx]

**Enhanced Emotional and Motor Responses to Live vs. Videotaped Dynamic Facial Expressions**

**Chun-Ting Hsu^1,^*, Wataru Sato^1,^*, Sakiko Yoshikawa^2^**

^1^Psychological Process Team, BZP, RIKEN, 2-2-2 Hikaridai, Seika-cho, Soraku-gun, Kyoto 619-0288, Japan.

^2^Institute of Philosophy and Human Values, Kyoto University of the Arts, 2-116 Uryuyama Kitashirakawa, Sakyo, Kyoto, Kyoto 606-8271, Japan.

* Corresponding Authors: hsuchunting@gmail.com; wataru.sato.ya@riken.jp

**Linear Mixed-effect Model Analysis of Non-transformed EMG Data**

We analyzed the EMG data without natural log(x+1) transformation to compare the results with results of transformed data. The same LME model structure was fit, the same criteria for outliers and highly influential data points exclusion was used. Subject No. 8 and 14 were excluded from the ZM analysis as in the log-transformed data, but for CS data, subject No. 13 and 14 were both identified as highly influential subjects.

Among ZM data, totally 226 trials (19.33%) were excluded. ZM results also showed a significant main effect of emotion, with greater muscle activation under the positive than under the negative conditions as well as an interaction between emotion and presentation condition (Supplementary Table S5, Figure S2A). Simple effect analysis showed greater muscle contraction in positive-live than positive-video condition (estimate = .054, SE = .0268, df = 61.8, t = 2.019, two-tailed p = .0478) but no difference between the negative-live and negative-video conditions (estimate = -0.0379, SE = .0269, df = 64.1, t = -1.41).

For the CS data, 277 trials (20.13%) were excluded. CS results showed a main effect of emotion, with the CS less active under the positive than under the negative conditions. The interaction is not significant (Supplementary Table S6, Figure S2B). Simple effect analysis showed a trend of less activity under the positive-live than under the positive-video condition (estimate = -0.1693, SE = .0838, df = 34.9, t = -2.021, two-tailed p = .051) but no difference between the negative-live and negative-video conditions (estimate = -0.0475, SE = .0832, df = 34, t = -0.571). As with log-transformed CS data, we also observed positive correlation between by-subject random slopes of the presentation condition and random slopes of emotion (Supplementary Table S6, 95% CI = 0.316, 1.161).

**Table S1 LME Analysis Summary of Validation Valence Ratings**

**Fixed Effects**

| **Effect** | **Beta** | **95% CI** | **SE** | **df** | **t-value** | **Pr(>\|t\|)** | **Rsq** |
| --- | --- | --- | --- | --- | --- | --- | --- |
| Intercept | 5.010 | (4.855, 5.164) | 0.0758 | 24.97 | 66.130 | < 2e-16 |  |
| Emotion | 2.547 | (2.002, 3.092) | 0.2676 | 25.01 | 9.517 | 8.58e-10 | 0.784 |
| Presentation | -0.106 | (-0.157, -0.054) | 0.026 | 163.8 | -4.069 | 7.31e-05 | 0.409 |
| Interaction | 0.00732 | (-0.062, 0.077) | 0.0354 | 1397 | 0.207 | 0.836 | < 0.001 |

**Random Effects**

| **Group** | **Effect** | **Variance** | **SD** | **SD 95% CI** | **Corr. I.** | **Corr. 95% CI** | **Corr. E.** | **Corr. 95% CI** |
| --- | --- | --- | --- | --- | --- | --- | --- | --- |
| Subject | Intercept | 0.136 | 0.368 | (0.28, Inf) |  |  |  |  |
|  | Emotion | 1.775 | 1.332 | (1.03, 1.81) | 0.10 | (-1, 1) |  |  |
|  | Presentation | 0.0012 | 0.034 | (0.011, 0.111) | -0.68 | (-0.837, 0.557) | 0.66 | (-0.805, 0.821) |
| Residual | | 0.4518 | 0.672 | (0.648, 0.698) |  |  |  |  |

Abbreviations: CI: confidence interval; Corr. I.: correlation with the random effect of intercept; Corr. E.: correlation with the random effect of emotion; df: Satterthwaite approximations to degrees of freedom; Rsq: effect size of semi-partial R-squared; SD: standard deviation; SE: standard error

**Table S2 LME Analysis Summary of Validation Arousal Ratings**

**Fixed Effects**

| **Effect** | **Beta** | **95% CI** | **SE** | **df** | **t-value** | **Pr(>\|t\|)** | **Rsq** |
| --- | --- | --- | --- | --- | --- | --- | --- |
| Intercept | 4.996 | (4.672, 5.319) | 0.159 | 25.04 | 31.447 | < 2e-16 |  |
| Emotion | 1.339 | (0.788, 1.889) | 0.27 | 24.95 | 4.956 | 4.19e-05 | 0.496 |
| Presentation | -0.117 | (-0.289, 0.053) | 0.084 | 23.31 | -1.398 | 0.175 | 0.073 |
| Interaction | 0.049 | (-0.054, 0.152) | 0.053 | 1404.28 | 0.93 | 0.352 | 0.001 |

**Random Effects**

| **Group** | **Effect** | **Variance** | **SD** | **SD 95% CI** | **Corr. I.** | **Corr. 95% CI** | **Corr. E.** | **Corr. 95% CI** |
| --- | --- | --- | --- | --- | --- | --- | --- | --- |
| Subject | Intercept | 0.613 | 0.783 | (0.602, 1.070) |  |  |  |  |
|  | Emotion | 1.789 | 1.337 | (1.031, 1.823) | -0.02 | (-0.404, 0.366) |  |  |
|  | Presentation | 0.141 | 0.375 | (0.261, 0.542) | 0.02 | (-0.407, 0.432) | -0.20 | (-0.577, 0.239) |
| Residual | | 1.019 | 1.009 | (0.973, 1.048) |  |  |  |  |

Abbreviations: CI: confidence interval; Corr. I.: correlation with the random effect of intercept; Corr. E.: correlation with the random effect of emotion; df: Satterthwaite approximations to degrees of freedom; Rsq: effect size of semi-partial R-squared; SD: standard deviation; SE: standard error

**Table S3 Bayesian and Frequentist Paired Sample T-tests for Validation Ratings**

**Paired T-tests between [Positive-live minus Negative-live] and [Positive-pre-recorded minus Negative-pre-recorded]**

| **Variables** | **BF_10_** | **BF_01_** | **Error %** | **Shapiro-Wilk W** | **p** | **Test** | **t** | **df** | **p** | **Mean Difference** | **SE Differenc** | **Mean 95% CI Lower** | **Mean 95% CI Upper** |
| --- | --- | --- | --- | --- | --- | --- | --- | --- | --- | --- | --- | --- | --- |
| **Valence** | **0.211** | **4.731** | **0.029** | **0.923** | **0.059** | **Student** | **0.075** | **24** | **0.941** | **0.01** | **0.134** | **-0.266** | **0.286** |
| **Valence M1** | **0.224** | **4.47** | **0.03** | **0.935** | **0.114** | **Student** | **-0.359** | **24** | **0.722** | **-0.07** | **0.195** | **-0.472** | **0.322** |
| **Valence M2** | **0.244** | **4.101** | **0.032** | **0.929** | **0.084** | **Student** | **0.565** | **24** | **0.577** | **0.09** | **0.159** | **-0.239** | **0.419** |
| **Arousal** | **0.211** | **4.738** | **0.029** | **0.951** | **0.261** | **Student** | **0.049** | **24** | **0.962** | **0.007** | **0.154** | **-0.31** | **0.325** |
| **Arousal M1** | **0.212** | **4.725** | **0.029** | **0.947** | **0.213** | **Student** | **0.091** | **24** | **0.928** | **0.02** | **0.219** | **-0.432** | **0.472** |
| **Arousal M2** | **0.211** | **4.742** | **0.029** | **0.981** | **0.91** | **Student** | **-0.026** | **24** | **0.979** | **-0.005** | **0.189** | **-0.396** | **0.386** |

**Descriptive Statistics**

| **Measures** | **N** | **Mean** | **SD** | **SE** | **Mean 95% CI Lower** | **Mean 95% CI Upper** |
| --- | --- | --- | --- | --- | --- | --- |
| **Valence Pre-recorded Differences** | **25** | **3.48** | **1.827** | **0.365** | **2.726** | **4.234** |
| **Valence Live Differences** | **25** | **3.47** | **1.81** | **0.362** | **2.723** | **4.217** |
| **Valence Pre-recorded Differences M1** | **25** | **3.115** | **1.859** | **0.372** | **2.348** | **3.882** |
| **Valence Live Differences M1** | **25** | **3.185** | **1.727** | **0.345** | **2.472** | **3.898** |
| **Valence Pre-recorded Differences M2** | **25** | **3.845** | **1.915** | **0.383** | **3.055** | **4.635** |
| **Valence Live Differences M2** | **25** | **3.755** | **1.966** | **0.393** | **2.943** | **4.567** |
| **Arousal Pre-recorded Differences** | **25** | **1.735** | **1.76** | **0.352** | **1.009** | **2.461** |
| **Arousal Live Differences** | **25** | **1.728** | **1.705** | **0.341** | **1.024** | **2.431** |
| **Arousal Pre-recorded Differences M1** | **25** | **1.19** | **1.896** | **0.379** | **0.407** | **1.973** |
| **Arousal Live Differences M1** | **25** | **1.17** | **1.539** | **0.308** | **0.535** | **1.805** |
| **Arousal Pre-recorded Differences M2** | **25** | **2.28** | **1.94** | **0.388** | **1.479** | **3.081** |
| **Arousal Live Differences M2** | **25** | **2.285** | **2.148** | **0.43** | **1.398** | **3.172** |

Footnotes: CI: confidence interval; df: Degrees of freedom; M1: model 1; M2: model 2; N: sample size of subject numbers; SD: standard deviation; SE: standard error

**Table S4 Bayesian and Frequentist One Sample T-tests for Pre-recorded/Live Performance Accuracy Rate**

**One Sample T-tests Against the Chance Level of 0.05**

| **Variables** | **BF_10_** | **BF_01_** | **Error %** | **Shapiro-Wilk W** | **p** | **Test** | **W** | **p** | **Hodges-Lehmann estimate** | **95% CI Lower** | **95% CI Upper** |
| --- | --- | --- | --- | --- | --- | --- | --- | --- | --- | --- | --- |
| **Accuracy** | **0.214** | **4.679** | **0.029** | **0.874** | **0.005** | **Wilcoxon** | **98.5** | **0.822** | **-0.008** | **-0.062** | **0.047** |
| **Accuracy M1** | **0.239** | **4.178** | **0.032** | **0.87** | **0.004** | **Wilcoxon** | **93** | **0.758** | **4.211e-5** | **-0.047** | **0.062** |
| **Accuracy M2** | **0.213** | **4.702** | **0.029** | **0.921** | **0.053** | **Wilcoxon** | **95** | **0.485** | **-0.016** | **-0.078** | **0.047** |

**Descriptive Statistics**

| **Measures** | **N** | **Mean** | **SD** | **SE** | **Mean 95% CI Lower** | **Mean 95% CI Upper** |
| --- | --- | --- | --- | --- | --- | --- |
| **Accuracy** | **25** | **0.504** | **0.109** | **0.022** | **0.459** | **0.549** |
| **Accuracy M1** | **25** | **0.511** | **0.107** | **0.021** | **0.467** | **0.555** |
| **Accuracy M2** | **25** | **0.496** | **0.137** | **0.027** | **0.44** | **0.553** |

Footnotes: CI: confidence interval; M1: model 1; M2: model 2; N: sample size of subject numbers; SD: standard deviation; SE: standard error; Wilcoxon: Wilcoxon signed-rank test. For the Wilcoxon test, the alternative hypothesis specifies that the median is different from 0.5.

**Table S5 Statistical Summary of Zygomaticus Major Reactions with Non-transformed Data**

**Fixed Effects**

| **Effect** | **Beta** | **95% CI** | **SE** | **df** | **t-value** | **Pr(>\|t\|)** | **Rsq** |
| --- | --- | --- | --- | --- | --- | --- | --- |
| Intercept | 0.059 | (0.008, 0.110) | 0.0249 | 20.44 | 2.375 | 0.02742 |  |
| Emotion | 0.1128 | (0.045, 0.181) | 0.0333 | 20.24 | 3.392 | 0.00286 | 0.354 |
| Presentation | 5.692e-03 | (-0.024, 0.035) | 0.0143 | 19.72 | 0.399 | 0.69438 | 0.008 |
| Interaction | 0.046 | (0.013, 0.079) | 0.017 | 1055 | 2.704 | 0.00697 | 0.007 |

**Random Effects**

| **Group** | **Effect** | **Variance** | **SD** | **SD 95% CI** | **Corr. I.** | **Corr. 95% CI** | **Corr. E.** | **Corr. 95% CI** |
| --- | --- | --- | --- | --- | --- | --- | --- | --- |
| Subject | Intercept | 0.0114 | 0.107 | (0.077, 0.155) |  |  |  |  |
|  | Emotion | 0.0201 | 0.142 | (0.101, 0.208) | 0.72 | (0.328, 0.924) |  |  |
|  | Presentation | 0.0012 | 0.035 | (0.0018, 0.074) | -0.26 | (-0.435, 1.000) | 0.09 | (-0.823, 0.403) |
| Residual | | 0.0799 | 0.283 | (0.271, 0.295) |  |  |  |  |

Abbreviations: CI: confidence interval; Corr. I.: correlation with the random effect of intercept; Corr. E.: correlation with the random effect of emotion; df: Satterthwaite approximations to degrees of freedom; Rsq: effect size of semi-partial R-squared; SD: standard deviation; SE: standard error

**Table S6 Statistical Summary of Corrugator Supercilii Reactions with Non-transformed Data**

**Fixed Effects**

| **Effect** | **Beta** | **95% CI** | **SE** | **df** | **t-value** | **Pr(>\|t\|)** | **Rsq** |
| --- | --- | --- | --- | --- | --- | --- | --- |
| Intercept | -0.2109 | (-0.342, -0.074) | 0.0685 | 19.39 | -3.082 | 0.00604 |  |
| Emotion | -0.2787 | (-0.444, -0.109) | 0.0869 | 20.32 | -3.209 | 0.00434 | 0.329 |
| Presentation | -0.0767 | (-0.185, 0.018) | 0.0516 | 18.01 | -1.486 | 0.15453 | 0.096 |
| Interaction | -0.0609 | (-0.132, 0.012) | 0.0375 | 1056.37 | -1.625 | 0.10442 | 0.003 |

**Random Effects**

| **Group** | **Effect** | **Variance** | **SD** | **SD 95% CI** | **Corr. I.** | **Corr. 95% CI** | **Corr. E.** | **Corr. 95% CI** |
| --- | --- | --- | --- | --- | --- | --- | --- | --- |
| Subject | Intercept | 0.091 | 0.301 | (0.212, 0.407) |  |  |  |  |
|  | Emotion | 0.143 | 0.378 | (0.266, 0.511) | 0.49 | (0.167, 0.965) |  |  |
|  | Presentation | 0.041 | 0.203 | (0.126, 0.286) | 0.99 | (0.978, 1.164) | 0.61 | (0.316, 1.161) |
| Residual | | 0.384 | 0.620 | (0.595, 0.648) |  |  |  |  |

Abbreviations: CI: confidence interval; Corr. I.: correlation with the random effect of intercept; Corr. E.: correlation with the random effect of emotion; df: Satterthwaite approximations to degrees of freedom; Rsq: effect size of semi-partial R-squared; SD: standard deviation; SE: standard error

**Supplementary Figure S1**


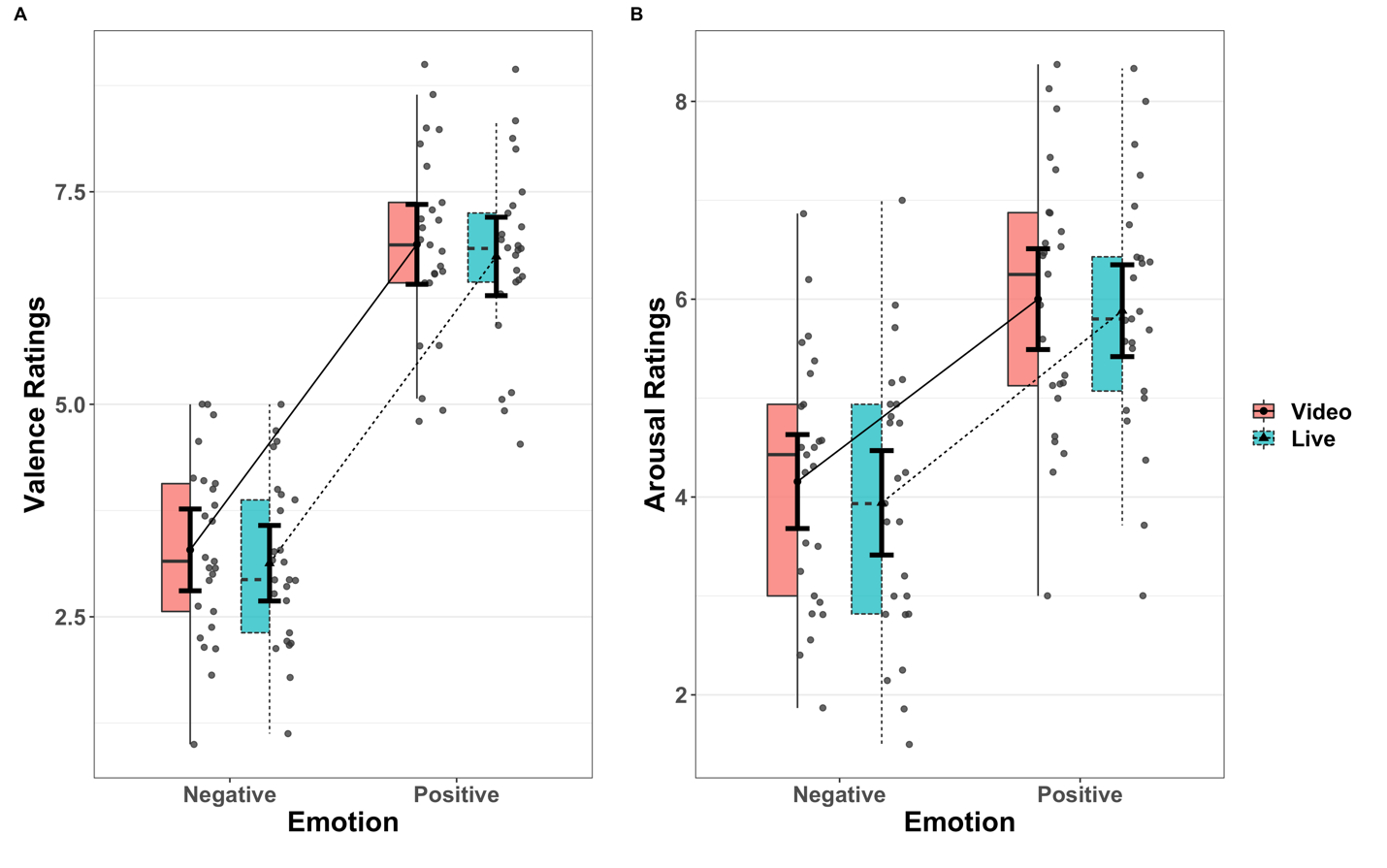


**Validation Rating Results.** For each condition, the right half showed the scattered dots of participant-wise mean values. The group mean value is shown with a filled dot (video condition) or triangle (live condition) in the middle accompanied by error bars of within-subject standard error. The box on the left half defined the median and the first and third quartiles of the distribution, and the upper or lower whisker extends from the hinge to the most extreme value no further than 1.5 * IQR from the hinge. Panel A: Valence. Panel B: Arousal. There is no interaction effect between emotion and presentation condition in the validation data.

**Supplementary Figure S2**


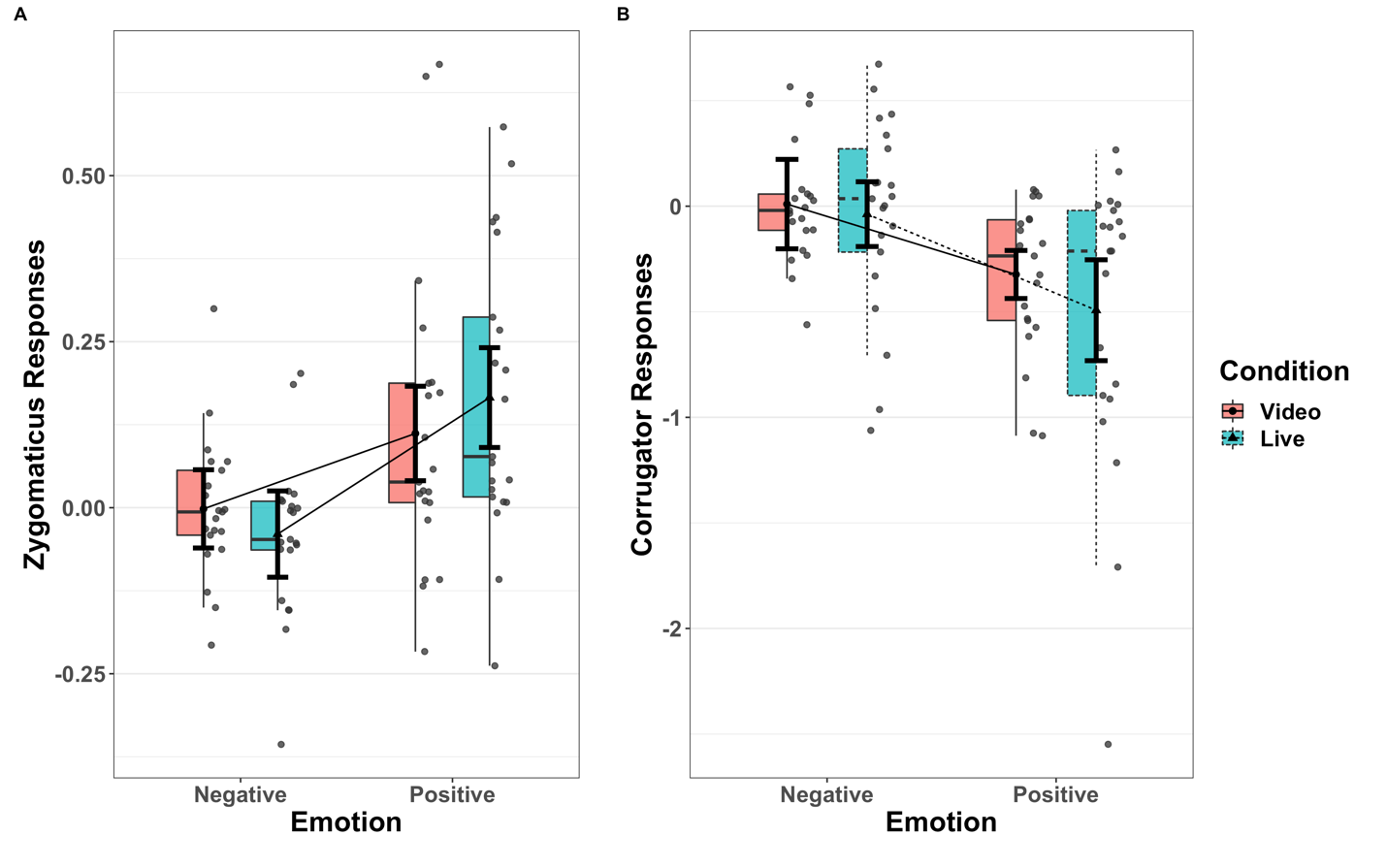


**Facial Electromyography (EMG) Results of Non-transformed Data.** Please see the legend of Figure S1 for component descriptions of the boxjitter plots. Panel A: Zygomaticus major (ZM). Panel B: Corrugator supercilii (CS). The ZM results showed a significant interaction between emotion (positive, negative) and presentation condition (video, live), which consisted of significant differences between the positive-live than the positive-video condition, but not between the negative conditions.
